# The Key Role of Non-Local Screening in the Environment-Insensitive Exciton Fine Structures of Transition-Metal Dichalcogenide Monolayers

**DOI:** 10.3390/nano13111739

**Published:** 2023-05-26

**Authors:** Wei-Hua Li, Jhen-Dong Lin, Ping-Yuan Lo, Guan-Hao Peng, Ching-Yu Hei, Shao-Yu Chen, Shun-Jen Cheng

**Affiliations:** 1 Department of Electrophysics, National Yang Ming Chiao Tung University, Hsinchu 300, Taiwan; 2 Center of Condensed Matter Sciences, National Taiwan University, Taipei 106, Taiwan; 3 Center of Atomic Initiative for New Material, National Taiwan University, Taipei 106, Taiwan

**Keywords:** transition-metal dichalcogenide monolayer, dark-exciton, non-local Coulomb screening, exciton fine structure, WSe2

## Abstract

In this work, we present a comprehensive theoretical and computational investigation of exciton fine structures of WSe${}_{2}$-monolayers, one of the best-known two-dimensional (2D) transition-metal dichalcogenides (TMDs), in various dielectric-layered environments by solving the first-principles-based Bethe–Salpeter equation. While the physical and electronic properties of atomically thin nanomaterials are normally sensitive to the variation of the surrounding environment, our studies reveal that the influence of the dielectric environment on the exciton fine structures of TMD-MLs is surprisingly limited. We point out that the non-locality of Coulomb screening plays a key role in suppressing the dielectric environment factor and drastically shrinking the fine structure splittings between bright exciton (BX) states and various dark-exciton (DX) states of TMD-MLs. The intriguing non-locality of screening in 2D materials can be manifested by the measurable non-linear correlation between the BX-DX splittings and exciton-binding energies by varying the surrounding dielectric environments. The revealed environment-insensitive exciton fine structures of TMD-ML suggest the robustness of prospective dark-exciton-based optoelectronics against the inevitable variation of the inhomogeneous dielectric environment.

## 1. Introduction

With exceptional valley and excitonic properties, atomically thin transition-metal dichalcogenide monolayers (TMD-MLs) have emerged as promising nanomaterials for valley-based optoelectronic and photonic applications [1,2,3,4,5,6]. Because of weak dielectric screening in the two-dimensional (2D) systems, photo-excited excitons in TMD-MLs are tightly bound by the enhanced electron–hole Coulomb interaction and featured with giant binding energies ranging between 200 and 500 meV [7,8,9,10], leading to superior light–matter interactions and various fascinating optical phenomena [11,12,13,14]. Even more interestingly, the pronounced Coulomb effects on those 2D excitons result in rich excitonic fine structures, which, typically by tens of meV, well-resolve the optically active bright exciton (BX) states as well as various dark-exciton (DX) ones. The latter are further classified by so-called spin-forbidden (SFDX) and momentum-forbidden dark-exciton (MFDX) states according to the violated optical selection rules [15,16,17,18,19,20]. Significant fine structure splitting of excitons enables a tremendously high exciton population in low-lying DX states and stabilizes the long-time residence of excitons [21], giving rise to intriguing dark-exciton-related optical phenomena, such as strong optical responses in nano-photonics [22,23,24,25], long-distance exciton diffusion [26,27], super-efficient energy transfer [13], Bose condensation [28,29], etc.

In general, the physical and chemical properties of ultra-thin 2D materials, such as TMD-MLs, are sensitive to the variation in dielectric environments. Following the conventional hydrogen model of excitons in the approximation of local dielectric screening [30,31], the binding energy of excitons at the scale of hundreds of meV is predicted to be inversely proportional to the quadratic dielectric constant ($\propto \varepsilon_{eff}^{-2}$), leading to the sensitive response of the exciton Rydberg series to the dielectric environments [32,33,34,35,36]. Experimentally, it has been established that the binding energy of excitons in TMD-MLs can be effectively controlled by engineering the dielectrics surrounding TMD-MLs [37,38,39,40,41], facilitating the feasibility of exciton-based devices where charge-neutral excitons need to be transported or spatially confined. On the other hand, there is a current desire to discover effective methods for controlling the fine structures of excitons in TMD-MLs at the meV scale, whose BX-DX splittings directly determine the population of long-lived dark excitons. Yet, the existing experiments reported in the literature do not observe the expected significant influences from dielectric environments on the exciton fine structures of TMD-MLs (See Table S2 of the supporting information and the references therein [21,22,42,43,44,45,46,47,48,49,50,51,52,53,54,55,56,57,58]), and the underlying physics remains puzzling [44].

In this work, we present a theoretical and computational investigation of the exciton fine structures of WSe${}_{2}$-MLs, composed of BX and DX states, by solving the first-principles-based Bethe–Salpeter-equation (BSE) [15], combined with the non-local dielectric functions of TMD-embedded multi-layered dielectric environments. Our studies reveal that the exciton fine structure splittings of WSe${}_{2}$-MLs under non-local Coulomb screening are essentially weakly dependent on the environment, which is different from what is expected from the conventional exciton theory with local dielectric constants. We point out the key role of non-locality in dielectric screening, which effectively suppresses the influence of the dielectric environment on the BX-DX fine structure splittings. The small Bohr radii of tightly bound excitons in 2D materials suggest the substantial spreading of exciton wave functions in the momentum space (*q*-space) and underline the essential role of *q*-dependent dielectric functions [8,40,59,60]. Moreover, we set up an extended exciton hydrogen model with the non-local dielectric function for the TMD-MLs embedded in complex multi-layered dielectric structures. The theoretical studies are supported by the quantitative agreement with our experimental measurements on the exciton fine structure splittings of WSe${}_{2}$-MLs and physically account for the commonly observed environment-insensitive dependence of exciton fine structure splittings in TMD-MLs [44].

## 2. Computational and Experimental Methods

### 2.1. First-Principles Calculations and Bethe–Salpeter Equation (BSE) for Exciton Spectra

The quasi-particle states of WSe${}_{2}$-ML in the density functional theory (DFT) were carried out by utilizing the first principles Vienna Ab initio Simulation Package (VASP) [61] with the Heyd–Scuseria–Ernzerhof (HSE) exchange-correlation functional [62] and the consideration of spin–orbit coupling (SOC). We employed the experimental lattice constant 3.282 Å [63,64] in the DFT calculation. While the lattice constant determined by the lowest total energy in the DFT calculation depends on the used exchange-correlation functional, in this work, we adopted the experimental lattice constant of the real sample in order to avoid the uncertainty due to the selection of the functional model [65]. The energy cutoff of the plane-wave basis was set at 500 eV for the expansion of the wave functions and the projector-augmented wave (PAW) potentials. The Γ-centered k-grid of 9×9×1 was taken to sample the Brillouin zone and the electronic relaxation convergence limit was set at $10^{-6}$ eV. In order to simulate the suspended WSe${}_{2}$ monolayer, we periodically placed TMD monolayers along the out-of-plane direction inserted with sufficiently thick vacuum layers to eliminate the unwanted coupling between TMD monolayers. According to the convergence test, we took the thickness of vacuum spacing to be 29.78 Å. In addition to the 3.38 Å-thick TMD monolayer, the height of the TMD-vacuum supercell in the DFT simulation was 33.16 Å. The convergence tests for the verifications of the numerical parameters used in the DFT calculations are given in the supporting information (See Figure S2–S4 for the calculation results).

**Figure 1:**
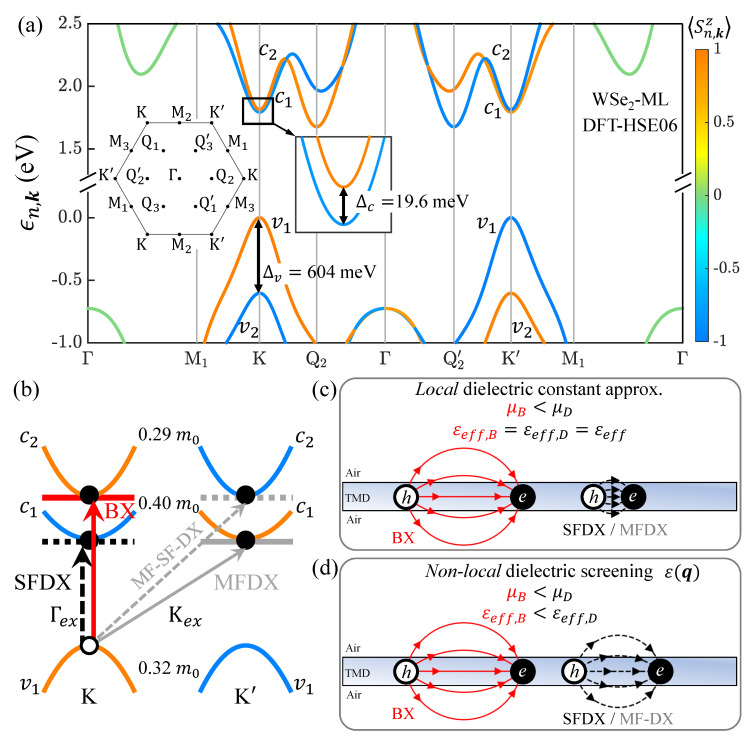
(**a**) Valley− and spin−resolved quasi-particle band structures of WSe${}_{2}$-ML calculated by using the HSE06 exchange-correlation functionals in the DFT [62]. The blue (orange) color stands for the spin-down (spin-up) Bloch states. Left inset: the first Brillouin zone of WSe${}_{2}$-ML, with the indication of high-symmetry points. Middle inset: the close-up of the lowest conduction bands around the *K* valley, with the spin-splitting, $\Delta_{c}=19.6$ meV. (**b**) Schematics of the intra-valley BX and SFDX, and the inter-valley MFDX and MF-SF-DX states, with the valence holes located at the *K* valley. (**c**) Schematics of BX and DX in a TMD-ML under a dielectric environment. In the approximation of local screening, the dielectric screening of the TMD-embedded layered system is simply characterized by a fixed dielectric constant $\varepsilon_{eff}$, which is the same for both BX and DX. Because of the heavier reduced mass of DX, the Bohr radius (binding energy) of DX is significantly smaller (larger) than the lighter BX. (**d**) Beyond the approximation of local screening, the non-local screenings for the BX and DX in the TMD-embedded layered system are described by the q-dependent dielectric function, ε(q), leading to the unequal effective screening for the BX and DX. The non-local screenings reduce the differences between the Bohr radii and binding energies of the BX and DX.

The exciton spectra were carried out by the theoretical methodology developed by Reference [15], which established an in-house BSE code based on the DFT-calculated quasi-particle band structures of 2D materials. Following the methodology used in Reference [15], we utilized the Wannier90 package [66,67] to transform the DFT-calculated Bloch functions into the set of maximally localized Wannier functions (MLWFs) and reconstruct the Kohn–Sham Hamiltonian in the Wannier tight-binding scheme. The calculated spin-resolved band structure of a WSe${}_{2}$-ML shown in Figure 1 is in excellent agreement with the one directly calculated by using DFT (See Figure S5 and Table S3 in the supporting information for the comparisons). Thus, the matrix elements of the Coulomb kernel of BSE are evaluated in terms of MLWFs obtained by utilizing the Wannier90 package [66,67]. Incorporating the interpolation techniques [15], the evaluation of the matrix elements of the Coulomb kernel can be carried out with high accuracy and low numerical costs.

### 2.2. Sample Fabrication and Experimental Photoluminescence (PL) Measurement

We exfoliated WSe${}_{2}$ from the bulk WSe${}_{2}$ crystal purchased from HQ Graphene. The WSe${}_{2}$-ML flakes were firstly exfoliated on a PDMS stamp and inspected under an optical microscope. The thickness of WSe${}_{2}$ is confirmed by optical contrast and optical spectroscopy. Then, we transferred the flakes to the desired substrates (e.g., SiO${}_{2}$, Al${}_{2}$O${}_{3}$, etc.) For hBN-encapsulated WSe${}_{2}$-ML samples, we followed the fabrication procedures of the PPC-assisted transfer mentioned in Reference [46]. The top and bottom hBNs were chosen to be around 20 nm to ensure good passivation and protection.

**Figure 2:**
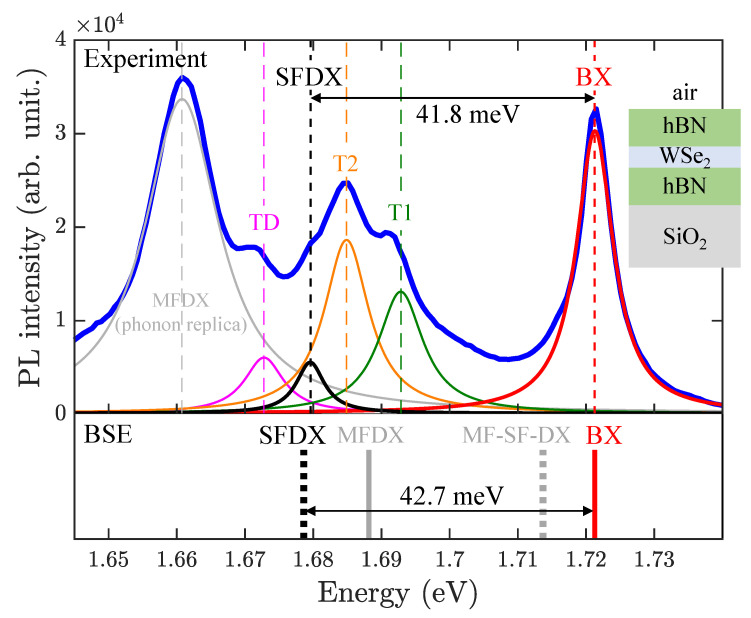
Comparison of the measured fine structure spectrum of PL at T=10 K (top panel) and BSE-calculated exciton fine structure (bottom panel) of hBN-encapsulated WSe${}_{2}$-ML. In the top panel, the measured PL spectrum is indicated by the blue profile. In the PL spectrum, the fitted peak by the red (black) profile is attributed to the BX (SFDX) state [46]. The BSE-calculated energy splitting of 42.7 meV between BX and SFDX states is in quantitative agreement with the fine structure of the measured 41.8 meV in the PL spectrum.

After fabrication, the samples were mounted in a Janis (ST-500) cryostat maintained at a high vacuum ($5\times 10^{-6}$ torr) and cooled down to 10 K by flowing liquid helium. For PL spectroscopy, we excited the sample with a CW laser at a wavelength of 532 nm. We employed a 40× objective lens (NA: 0.6) to focus the laser, achieving a spot size of about ∼1 μm on the sample, and collecting the PL signal in backscattering geometry. The signal was guided to the Horiba iHR 550 spectrometer and then detected by the liquid nitrogen-cooled charge-coupled device from the Horiba Symphony II detection system. The experimental PL spectrum of a hBN-encapsulated WSe${}_{2}$-ML is shown in Figure 2.

## 3. Results and Discussion

### 3.1. Quasi-Particle Band Structures of the WSe${}_{2}$ Monolayer

Figure 1a presents the quasi-particle electronic band structures of the WSe${}_{2}$ monolayer by using the methodology presented in Section 2.1. The bands are presented in colors according to the average of the z-component of the spin, $\langle S_{n,k}^{z}\rangle =\langle \psi_{n,k}\left|{\hat{\sigma }}_{z}\right|\psi_{n,k}\rangle$, where $|\psi_{n,k}\rangle$ is the Bloch function of the *n*-th band with the wave vector k, and ${\hat{\sigma }}_{z}$ is the Pauli matrix for the z-component of the spin. As this work is focused on the low-lying spectra of the A-exciton, we shall pay attention to the low-lying conduction and topmost valence bands, as shown in Figure 1a. Due to the effect of SOC, the conduction [valence] bands at the *K* and $K^{\prime }$ valleys are split by $\Delta_{c}\left(K/K^{\prime }\right)\equiv \epsilon_{c_{2},K/K^{\prime }}-\epsilon_{c_{1},K/K^{\prime }}\approx 19.6\;\mathrm{meV}>0$ [$\Delta_{v}\left(K/K^{\prime }\right)\equiv \epsilon_{v_{1},K/K^{\prime }}-\epsilon_{v_{2},K/K^{\prime }}\approx 604$ meV], which leads also to the distinct effective masses of the spin-split conduction bands in the *K* and $K^{\prime }$ valleys, as indicated in Figure 1b and Table 1. Since the lowest spin-split conduction band possesses the opposite spin to that of the topmost valence band in the same valley, the lowest exciton states of WSe${}_{2}$-ML are expected to be SFDX states. In addition to $\Delta_{c}$, the Coulomb interactions, both direct and exchange ones, of excitons substantially affect the BX-DX fine structure splittings of WSe${}_{2}$-MLs, as will be shown later.

### 3.2. Theory of Exciton Fine Structures of TMD-MLs under Dielectric Screenings

To compute the exciton spectra, we employed the theoretical methodology developed by Reference [15], which establishes the BSE on the first-principles base and numerically solves the exciton fine structures in an efficient manner. The BSE governing the exciton wave function and energy spectra consists of the kinetic energies of electron–hole pairs and the *e-h* Coulomb kernel, which consists of the attractive screened *e-h* direct interaction and the repulsive exchange one [15]. The screened direct Coulomb interaction is associated with the non-local dielectric function of a TMD-ML surrounded by a specific dielectric environment that can be solved by using the classical electromagnetic theory. The precise evaluation of the matrix elements of the Coulomb kernel that essentially involves the rapidly varying microscopic parts of the Bloch wave functions is computationally non-trivial. In the Wannier tight-binding scheme, the evaluation of the electron–hole exchange interactions in terms of Wannier functions can be carried out with significantly reduced numerical costs [15].

Following Reference [32], the 2D Fourier transform of the screened direct Coulomb interaction $W\left(q;z_{1},z_{2}\right)$ is obtained by solving Poisson’s equation, which reads

(1) $$\left[-q^{2}\varepsilon_{q}\left(z_{1}\right)+\frac{\partial }{\partial z_{1}}\varepsilon_{q}\left(z_{1}\right)\frac{\partial }{\partial z_{1}}\right]W\left(q;z_{1},z_{2}\right)=-\frac{e^{2}}{\varepsilon_{0}}\delta \left(z_{1}-z_{2}\right)\;,$$

where $q=(q_{x},q_{y})$ is the in-plane wave vector, *z* is the out-of-plane coordinate, $\varepsilon_{q}\left(z\right)$ is the *z*-dependent macroscopic dielectric function for the dielectric system consisting of the TMD-ML and the surrounding dielectric layers. For the dielectric layers, $\varepsilon_{q}\left(z\right)$ is considered to be piecewise constant (See Equation (S6) in the supporting information). For the sandwiched TMD-ML itself, we adopted the non-local dielectric functions and the parameters for WSe${}_{2}$-ML given by References [68,69].

**Figure 3:**
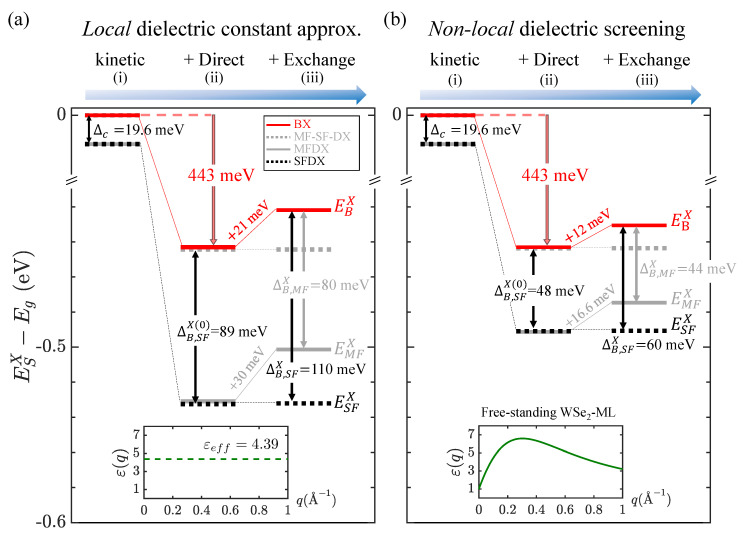
The BSE−calculated low-lying exciton fine structures of free-standing WSe${}_{2}$-ML (**a**) with fixed $\varepsilon_{eff}=4.39$ in the approximation of local screening and (**b**) with the q-dependent non-local dielectric function, ε(q) (see the inset at the bottom), beyond the approximation of local screening. (i–iii) in (**a**,**b**): The exciton fine structure spectra calculated by solving the modified or exact BSE that take into account (i) only the kinetic energies, (ii) the kinetic energies and the direct Coulomb interaction, and (iii) all of the kinetic energies and both the direct and exchange Coulomb interactions, respectively. From the evolution of the spectra from (i), (ii), and (iii), the *e-h* direct interaction is the dictating mechanism of the BX-SFDX splitting. Comparing (**a**,**b**), the non-local screening is shown to make the overall shrinkage of the fine structure splittings between the BX and various DXs.

By solving Equation (1), we can solve the effective non-local dielectric functions for various TMD-ML-embedded multi-layer systems with the number of dielectric layers, i.e., up to five (see Equation (S15) and Figure S1). Taking the *z*-average of $W\left(q;z_{1},z_{2}\right)$ over the thickness of TMD-ML [32], the Fourier transform of the *z*-average of the screened Coulomb interaction is given by $W\left(q\right)\approx \frac{1}{d^{2}}\int_{-d/2}^{d/2}\int_{-d/2}^{d/2}dz_{1}dz_{2}\;W\left(q;z_{1},z_{2}\right)$. Likewise, $V\left(q\right)=\;\frac{1}{d^{2}}\int_{-d/2}^{d/2}dz_{1}\int_{-d/2}^{d/2}dz_{2}$$\times \int d^{2}\rho \;V\left(\rho ,z_{1}-z_{2}\right)e^{-iq\cdot \rho }=\frac{e^{2}}{4\pi \varepsilon_{0}}\;\frac{4\pi }{dq^{2}}\left(1-\frac{1-e^{-qd}}{qd}\right)$ is the *z*-average of the bare Coulomb interaction. Accordingly, one can evaluate the non-local dielectric function defined by $\varepsilon \left(q\right)=V\left(q\right)/W\left(q\right)$ [10,70]. In this work, we assume that the non-local dielectric function is isotropic and depends only on the magnitude of q. Incorporating the solved $\varepsilon \left(q\right)$ for a free-standing WSe${}_{2}$-ML in the Coulomb kernel of BSE, the exciton fine structure of a free-standing WSe${}_{2}$-ML under non-local screening is numerically calculated by solving the BSE, as shown in Figure 3b.

**Figure 4:**
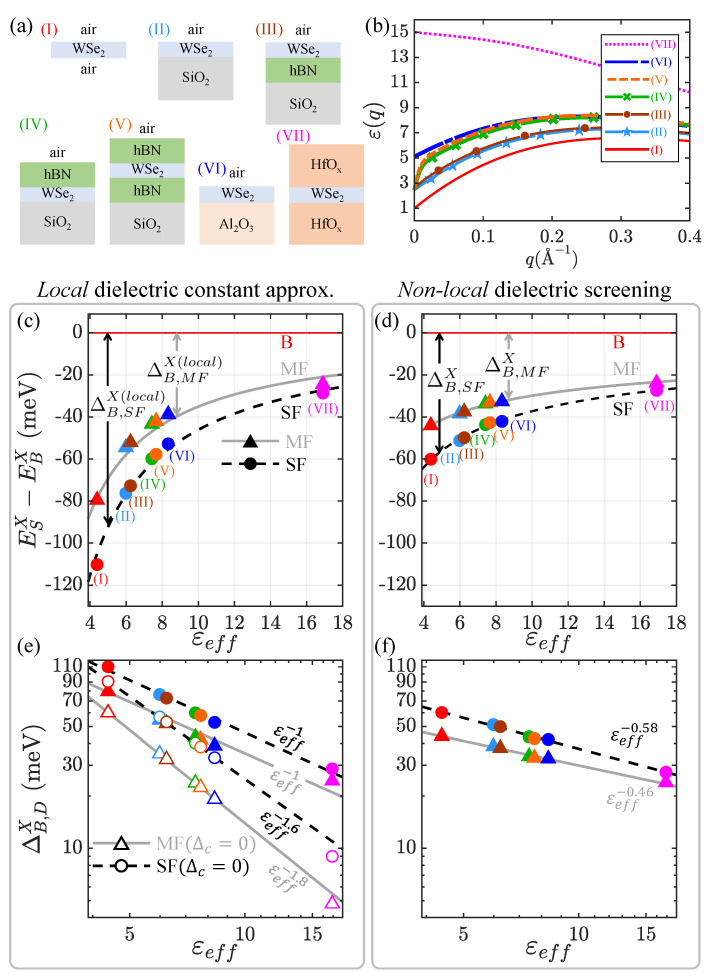
(**a**) The WSe${}_{2}$-ML−embedded dielectric environments considered in this work. In cases (I)–(VII), all of the substrate and capping layers are considered semi-infinitely thick, except for the hBN layer, whose thickness is set at 10 nm. (**b**) The calculated *q*-dependent dielectric functions for dielectric systems (I)–(VII). (**c**) The calculated relative energy levels of the BX, MFDX, and SFDX states of the WSe${}_{2}$-ML under the dielectric screenings of (I)–(VII), indicated by filled triangles (MFDX) and circles (SFDX) in different colors, in the approximation of local screening with the fitted dielectric constants, $\varepsilon_{eff}$. Dark dashed and gray solid curves are for eye-guiding; (**d**) is the same as (**c**), but under the non-local dielectric screening described by ε(q). (**e**) [(**f**)]: The log–log plot for the BX-DX splittings, $\Delta_{B,D}^{X}=E_{B}^{X}-E_{D}^{X}$, given by (**c**) [(**d**)], fitted by the power law of $\Delta_{B,D}^{X}\propto \varepsilon_{eff}^{-n}$ with the exponent *n*. The white-filled symbols in (**e**) are the calculated splittings, disregarding $\Delta_{c}$. Taking into account the non-local screening, the exponent is decreased to the small values of n∼0.58,0.46, indicating the weak environmental dependence of the BX-DX splitting.

To confirm the validity of the theoretical prediction, we performed the cryogenic photoluminescence (PL) measurement on the sample of hBN-encapsulated WSe${}_{2}$-ML (corresponding to case V of Figure 4) and compared the measured optical spectrum with the BSE-calculated fine structures of BX and DX. The experimental details are given in Section 2.2. As shown in the top panel of Figure 2, a typical PL spectrum at 10 K exhibits multiple sharp features. The peak with the highest energy is the BX; the rest are associated with dark excitons and other bound excitonic states. To extract the energies of these features, we performed multi-peak fitting with Lorentzian functions. Based on the assignments of the PL peaks in Reference [46], the fitted peak by the red (black) profile is attributed to the BX (SFDX) state. In the bottom panel, the vertical lines indicate the BSE-calculated energies of the BX (solid red) and SFDX (dot black), in quantitative agreement with the fine structure of the measured PL spectrum. The other fitted peaks in the PL spectrum are attributed to the states of the inter-valley negatively charged trion (T1), the intra-valley negatively charged trion (T2), and the exciton–trion five-particle state (TD) [46].

The computed exciton fine structures with full consideration of the Coulomb interactions and the non-local dielectric function show the splitting between the BX and SFDX states of hBN-encapsulated WSe${}_{2}$-ML to be 42.7 meV, in excellent agreement with the measured BX-SFDX splitting, 41.8 meV, shown in Figure 2. In addition to the case of hBN-encapsulated WSe${}_{2}$-ML, the exciton fine structures of WSe${}_{2}$-MLs with different surrounding dielectric materials are also calculated and shown consistent with the measured splittings reported in the literature, as summarized in Table S2.

### 3.3. Exciton Fine Structures in the Approximation of *Local* Screening

For a systematic investigation, we first purposely disregard the non-locality of screening and replace the *non-local q*-dependent dielectric function with a fitted effective dielectric constant, $\varepsilon_{eff}$, in the Coulomb kernel of the BSE. In the approximation of local screening, Figure 3a shows the calculated low-lying fine structure of the exciton for a free-standing WSe${}_{2}$-ML with the effective dielectric constant $\varepsilon_{eff}=4.39$ in the modified BSE that takes into account (i) only the kinetic energies, (ii) the kinetic energies and screened direct Coulomb interaction, and (iii) the kinetic energies and the full Coulomb interactions, including the direct and exchange ones, respectively. The effective dielectric constant $\varepsilon_{eff}=4.39$ is determined by fitting the numerically calculated binding energy of BX, $E_{B}^{b}=443$ meV, for a free-standing WSe${}_{2}$-ML to the $\varepsilon_{eff}$-parametrized 2D hydrogen model [30,31].

In the 2D exciton hydrogen model, the energy of the exciton in the *S*-state (S=B,SF,MF,...) is explicitly given by $E_{S}^{X(0)}=E_{g}-4Ry\left(\frac{\mu_{S}}{m_{0}}\right)\varepsilon_{eff}^{-2}$, with the binding energy of the exciton,

(2) $$E_{S}^{b}\equiv E_{g}-E_{S}^{X(0)}=4Ry\left(\frac{\mu_{S}}{m_{0}}\right)\varepsilon_{eff}^{-2}\;,$$

where Ry=13.6 eV is the Rydberg constant and $\mu_{S}={\left(m_{c}^{-1}+m_{v}^{-1}\right)}^{-1}$ is the reduced mass of the exciton. For WSe${}_{2}$-ML, $\mu_{B}=0.15\;m_{0}$ and $\mu_{SF}=0.18\;m_{0}$ (see Table 1).

Taking only the kinetic energies of the electron and hole, the non-interacting exciton fine structures shown in Figure 3(a-i) are simply featured with two multi-fold degenerate levels split by $\Delta_{c}=19.6$ meV. Turning on the direct Coulomb interaction, as shown in Figure 3(a-ii), the degeneracies of the exciton states remain the same, but the BX-SFDX splitting $\Delta_{B,SF}^{X(0)}\equiv E_{B}^{X(0)}-E_{SF}^{X(0)}$ is significantly enlarged, from 19.6 to 89 meV. Hereafter, we use the superscript (0) to denote the exchange-free exciton states subjected only to the attractive *e-h* direct interaction, and the abbreviated subscripts, *B*, SF, and MF, to denote BX, SFDX, and MFDX, respectively. The unequal reduced masses of BX and SFDX, $\mu_{B}<\mu_{SF}$, result in the different Bohr radii, $a_{B}^{X}>a_{SF}^{X}$, which are defined by $a_{S}^{X}\equiv \left(\frac{4\pi \epsilon_{0}\hbar^{2}}{e^{2}}\right)\cdot \left(\frac{\varepsilon_{eff}}{\mu_{S}}\right)$. For a free-standing WSe${}_{2}$-ML with $\varepsilon_{eff}=4.39$, $a_{B}^{X}=1.5$ nm ($a_{SF}^{X}=1.3$ nm) for BX (SFDX) with the reduced mass, $\mu_{B}=0.15\;m_{0}$ ($\mu_{SF}=0.18\;m_{0}$) is determined. With the smaller Bohr radius, the heavier SFDX gains more *e-h* Coulomb attraction and acquires a larger exciton-binding energy than the lighter BX (see Figure 1c for the schematic illustration). According to Equation (2), the unequal binding energies of the BX and SFDX states ($E_{B}^{b}<E_{SF}^{b}$) increase the BX-SFDX energy splitting by

(3) $$\Delta_{B,SF}^{X(0)}=\Delta_{c}+\frac{4Ry}{m_{0}\varepsilon_{eff}^{2}}\cdot \left(\mu_{SF}-\mu_{B}\right)\;,$$

evaluated as 89 meV, which is several times greater in magnitude than $\Delta_{c}=19.6$ meV.

Further taking the *e-h* exchange interaction into account, as shown in Figure 3 (a-iii), the energy of BX states is raised by the short-ranged (SR) *e-h* exchange interaction, which is numerically calculated as $V_{B}^{x}=E_{B}^{X}-E_{B}^{X(0)}=21$ meV, while the energies of the lowest SFDX states, which are free of exchange interactions, remain unchanged. Thus, under the approximation of the *local* screening with the fixed $\varepsilon_{eff}$, the BX-SFDX splitting is predicted to be $\Delta_{B,SF}^{X}\equiv E_{B}^{X}-E_{SF}^{X}=110$ meV, which is much larger than the measured splitting in the range of ∼50 meV [21,22,23,42,43,47,48,49,52,53,54,58,71]. In fact, as will be shown later, the non-locality in dielectric screening neglected so far is essential in the exciton fine structures; this non-locality drastically reduces the splitting to fall into the experimental range.

### 3.4. Exciton Fine Structure under *Non-Local* Dielectric Screening

Figure 3b shows the calculated exciton fine structure of a free-standing WSe${}_{2}$-ML by solving the BSE with the *non-local* dielectric function, ε(q), shown by the lower inset in Figure 3b. In comparison with Figure 3(a-iii), the BX-DX splitting, $\Delta_{B,SF}^{X}$, of the free-standing WSe${}_{2}$-ML under the *non-local* dielectric screening, as shown in Figure 3(b-iii), is drastically decreased from 110 to 60 meV. Overall, the fine structure splittings are shrunk by the decreases in the direct- and exchange-interaction-induced splittings due to the non-local effect of screening. The decrease in the BX-DX splitting occurs because the Bohr radius (binding energy) of the heavier DX state is increased (decreased) by the non-local screening that is more than the lighter BX state, as illustrated by Figure 1c,d. In other words, the effective dielectric constant of the DX state turns out to be greater than that of BX, i.e., $\varepsilon_{eff,D}=\varepsilon_{eff,B}+\Delta \varepsilon$ with Δε>0, considering the non-locality of screening.

With a given *q*-dependent dielectric function, ε(q), the effective dielectric constant of an exciton in the *S* state (S=B,SF,MF,...) can be estimated by [72]

(4) $$\varepsilon_{eff,S}\approx 2{\left(\frac{a_{S}^{X}}{2}\right)}^{2}\int_{0}^{2/a_{S}^{X}}dq\;q\varepsilon \left(q\right)\;.$$

The formalism of Equation (4) suggested by Reference [72] is based on the fact that the major part of the exciton wave function is localized in the finite reciprocal q-space that is roughly enclosed by the circle of the radius ∼$2/a_{S}^{X}$. The large binding energy of an exciton in TMD-ML leads to a small Bohr radius of a few nm, suggesting a significant spreading of the exciton wave function in the *q*-space. Therefore, a *q*-dependent non-local dielectric function is critical to capture the microscopic difference between different kinds of excitonic complexes.

To determine Δε in an analytical manner, we extend the conventional hydrogen model combined with the non-local dielectric function,

(5) $$\varepsilon \left(q\right)\approx c_{0}+c_{1}q+c_{2}q^{2}+...\;,$$

expanded in a Taylor series for small $q\in \{0,2/a_{S}^{X}\}$, where $c_{0}{\equiv \varepsilon \left(q\right)|}_{q=0}$, $c_{1}{\equiv \partial \varepsilon \left(q\right)/\partial q|}_{q=0}$, and $c_{2}\equiv \frac{1}{2}\partial^{2}\varepsilon \left(q\right)/\partial q^{2}{|}_{q=0}$. Taking $a_{S}^{X}=\left(\frac{4\pi \varepsilon_{0}\hbar^{2}}{e^{2}}\right)\left(\frac{\varepsilon_{eff,S}}{\mu_{S}}\right)$ as a function of $\varepsilon_{eff,S}$ in Equation (4), one can solve Equation (4) for $\varepsilon_{eff,S}$ in a iterative manner.

In the first-order approximation, where $\varepsilon \left(q\right)\approx c_{0}+c_{1}q$, the difference between $\varepsilon_{eff,D}$ and $\varepsilon_{eff,B}$ is derived from Equation (4) as

(6) $$\begin{array}{cc} \Delta \varepsilon \equiv \varepsilon_{eff,D}-\varepsilon_{eff,B} & =\frac{1}{2}\sqrt{c_{0}^{2}+\frac{4e^{2}c_{1}}{3\pi \varepsilon_{0}\hbar^{2}}\mu_{D}}-\frac{1}{2}\sqrt{c_{0}^{2}+\frac{4e^{2}c_{1}}{3\pi \varepsilon_{0}\hbar^{2}}\mu_{B}}\;. \end{array}$$

With $\mu_{D}>\mu_{B}$, Equation (6) shows Δε>0, accounting for the greater effective dielectric constant of the heavier DX than the lighter BX.

**Figure 5:**
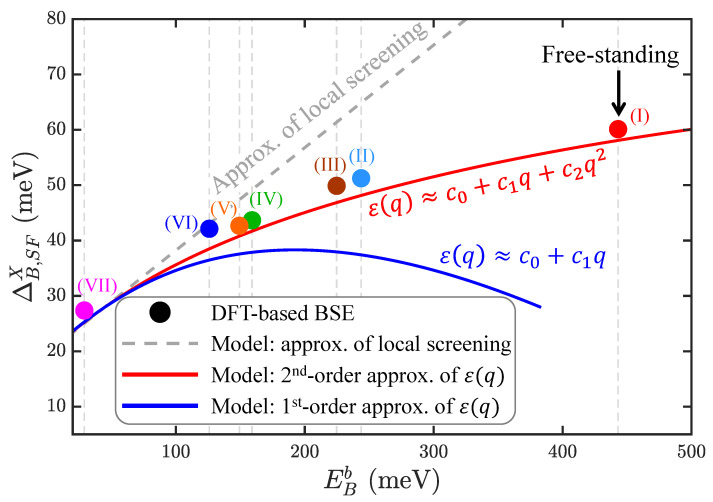
The non-linear correlation between the BX-SFDX splittings ($\Delta_{B,SF}^{X}$) and binding energies of BX ($E_{B}^{b}$) of WSe${}_{2}$-ML, changing the dielectric environments from (I) to (VII) in Figure 4a. The colored circles were obtained by solving DFT-based BSE for the WSe${}_{2}$-MLs in the multi-layered dielectric structures with full consideration of the non-local dielectric functions. The gray dashed line is the predicted linear $\Delta_{B,SF}^{X}$−$E_{B}^{b}$ by the conventional hydrogen model in the approximation of local screening. The red and blue solid lines are the non-linear correlations simulated by the extended hydrogen model with the expanded non-local dielectric functions in the first- and second-order approximations of ε(q), respectively. The sub-linear correlation between $\Delta_{B,SF}^{X}$ and $E_{B}^{b}$ manifests the insensitive environmental dependence of the exciton fine structures as the consequence of non-local dielectric screening.

For better quantitative studies, one can expand $\varepsilon \left(q\right)\approx c_{0}+c_{1}q+c_{2}q^{2}$ up to the second-order term, which has been shown to provide results that are in good agreement with the numerical ones, as shown in Figure 5. For symmetric three-layered structures, i.e., a TMD-ML sandwiched by two semi-infinite dielectrics, the *q*-dependent dielectric functions expanded in the Taylor series are solvable and considered in the model analysis throughout this work (see the supporting information for more details). In the second-order approximation, Δε=0.18 is estimated for a free-standing WSe${}_{2}$-ML. Taking Δε=0.18, the calculated BX-SFDX splitting of a free-standing WSe${}_{2}$-ML is decreased from 122 to 62 meV, which is in agreement with the numerically calculated splitting of 60 meV.

### 3.5. Dielectric Environmental Dependencies of Exciton Fine Structures

Now, let us investigate the influence of the dielectric environment on the exciton fine structures of TMD-MLs with the full consideration of non-local dielectric screening. Table S2 in the supporting information summarizes (from the literature) the experimentally measured fine structure splittings between BX and SFDX states of WSe${}_{2}$ monolayers in the dielectric environments of different materials and structures, which consistently fall into a narrow energy range between 40 and 50 meV for various dielectric environments, and show weak environmental dependencies [44]. For realistic simulations, we consider WSe${}_{2}$-MLs under various complex dielectric environments in the multi-layered structures composed of (I) air/WSe${}_{2}$/air (free-standing TMD-ML), (II) air/WSe${}_{2}$/SiO${}_{2}$, (III) air/WSe${}_{2}$/hBN/SiO${}_{2}$, (IV) air/hBN/WSe${}_{2}$/SiO${}_{2}$, (V) air/hBN/WSe${}_{2}$/hBN/SiO${}_{2}$, (VI) air/WSe${}_{2}$/Al${}_{2}$O${}_{3}$, and (VII) HfO${}_{x}$/WSe${}_{2}$/HfO${}_{x}$, as depicted in Figure 4a. In the layered structures of (I)–(VII), all of the substrate and capping layers are considered semi-infinitely thick, except for the hBN layer, whose thickness is set at 10 nm. The dielectric constants of the dielectric materials of the layered structures (I)–(VII) are shown in Table S1.

Figure 4d shows the exciton fine structure spectra of BX, inter-valley MFDX, and intra-valley SFDX states of the WSe${}_{2}$-MLs in the dielectric environments of Figure 4a, calculated by solving the BSE with the full non-local dielectric functions ε(q), versus the fitted effective dielectric constants $\varepsilon_{eff}$ for the screened TMD systems. By fitting the numerically calculated binding energy of BX to the formalism of the $\varepsilon_{eff}$-parametrized hydrogen model, $\varepsilon_{eff}$ = 4.39, 5.99, 6.24, 7.42, 7.66, 8.33, and 16.9, are determined for cases (I)–(VII) in Figure 4a, respectively. To underline the non-local effect of screening, Figure 4c shows the calculated exciton fine structure spectra of the dielectric-surrounded WSe${}_{2}$-MLs by solving the BSE with the fixed constant of $\varepsilon_{eff}$ for comparison.

Figure 4e is the log–log plot, based on the data of Figure 4c in the approximation of local screening, of the calculated BX-DX splittings of the screened WSe${}_{2}$-MLs versus $\varepsilon_{eff}$. It is shown that the calculated BX-DX splittings of WSe${}_{2}$-ML under local screenings follow the $\varepsilon_{eff}$-power law, $\Delta_{B,D=SF,MF}^{X}\propto \varepsilon_{eff}^{-n}$, with the exponent n≈1. The value of the exponent n∼1 is smaller than that of the power law for the exciton-binding energy, i.e., n=2 as shown by Equation (2), because the $\varepsilon_{eff}$-independent $\Delta_{c}$ takes part in the BX-DX splitting. Artificially removing $\Delta_{c}$ from the calculation, the $\Delta_{c}$-free BX-SFDX (BX-MFDX) splitting shows the $\varepsilon_{eff}$-power law with the n≈1.6 (n≈1.8), which is very close to n=2 given by the conventional hydrogen model.

Figure 4f is the log–log plot of the calculated BX-DX splittings of WSe${}_{2}$-MLs based on the data of Figure 4d with the consideration of non-local screening, showing the power law of $\Delta_{B,SF}^{X}$ in $\varepsilon_{eff}$ with n≈0.58. The very small value of *n* indicates the relatively weak $\varepsilon_{eff}$ dependencies of the BX-DX splitting of WSe${}_{2}$-MLs and accounts for the experimental observations of Table S2 and Reference [44]. The weak $\varepsilon_{eff}$ dependence of BX-DX splitting is explicitly shown by Equation (S28) as a consequence of non-local dielectric screening, which decreases the BX-DX splitting by the cubic $\varepsilon_{eff}^{-3}$ term, $\Delta_{B,D}^{X(non-loc.)}=-8\varepsilon_{eff}^{-3}\Delta \varepsilon \left(\mu_{D}/m_{0}\right)Ry$, which is associated with the non-zero Δε that reflects $\mu_{D}\neq \mu_{B}$, as illustrated by Figure 1c.

### 3.6. Non-Linear Correlation between the BX-DX Splitting and Exciton-Binding Energy under Non-Local Screening

The non-locality in dielectric screening can be manifested by the non-linear correlation between the BX-DX splitting and binding energy with varying $\varepsilon_{eff}$. In the approximation of local screening, Equations (2) and (3) show that both the BX-DX splitting and the binding energy of an exciton in a TMD-ML is dominated by the $\varepsilon_{eff}^{-2}$ terms. Beyond the approximation of local screening, the non-locality-induced $\varepsilon_{eff}^{-3}$ term with a negative sign in Equation (S28) takes part in the BX-DX splitting and makes the correlation between the BX-DX splitting and exciton-binding energy non-linear.

Figure 5 presents the scatter plot of the numerical BSE-calculated BX-SFDX splittings versus the exciton-binding energies of WSe${}_{2}$-MLs in the multi-layered dielectric structures (I)–(VII) shown in Figure 4a, considering the non-local dielectric functions. As expected from the model analysis, the BSE-calculated fine structure splittings and binding energies of exciton with varying $\varepsilon_{eff}$ are shown to be non-linearly correlated, i.e., following a sub-linear relationship.

Beyond the approximation of local screening, the extended hydrogen model combined with the approximate non-local ε(q) expanded up to the first-order term in Equation (5) predicts the blue curve in Figure 5. It is clearly seen that, by introducing the *q*-dependence of the dielectric function, the BX-SFDX splitting becomes weakly correlated with the binding energy of the exciton. Yet, compared with the numerical data, the model simulation in the first-order approximation of ε(q) overestimates the non-linear correlation between the BX-SFDX splitting and binding energy of the exciton. The validity of the exciton model with non-local screening can be improved by taking the non-local dielectric function, expanding up to the second-order term, and solving the Δε more precisely (See Equation (S21)). The model-simulated results with the non-local dielectric function in the second-order approximation is in excellent agreement with the numerical BSE-calculated data.

## 4. Conclusions

In summary, we carried out a comprehensive theoretical investigation, implemented on first-principles calculations, of the exciton fine structures of WSe${}_{2}$-ML under dielectric screenings from complex dielectric-layered environments. While the physical and electronic properties of atomically thin 2D materials are generally sensitive to the variation of surrounding environments, our first-principles-based theoretical studies, justified by the agreement with the measured optical spectra, point out that the non-locality of dielectric screening plays a key role in suppressing the influence of the dielectric environment on the exciton fine structure spectra of 2D materials. The theoretical studies account for the weak environmental dependencies of exciton fine structure splittings in TMD-MLs commonly observed in the existing experiments. In addition to the full numerical studies, we set up an extended exciton hydrogen model with the non-local dielectric function, successfully enabling the quantitative simulation and analysis of the exciton fine structures of TMD-MLs surrounded by complex multi-layered dielectrics. The intriguing non-locality in dielectric screening is manifested by the measurable non-linear correlation between the BX-DX splittings and exciton-binding energies, which vary with the effective dielectric constant of the environment. Despite the poor tunability of the exciton fine structure through engineered dielectric environments, the revealed weak environmental dependencies of exciton fine structures suggest the robustness of dark-exciton-based physical properties against the variations or fluctuations of dielectric environments.

## Figures and Tables

**Table 1:** Effective masses of the conduction electrons and valence holes in the *K*- or *Q*-valleys, the reduced masses of the bright exciton ($\mu_{B}$), and that of the spin-forbidden dark exciton ($\mu_{SF}$) in the free electron mass $m_{0}$ unit. The method for determining the effective mass is detailed in the supporting information (See Figure S6 and Table S4 for the fitting results).

Effective Masses of Carriers ($m_{0}$)				Reduced Masses of Excitons ($m_{0}$)	
$m_{v_{1},K}$	$m_{c_{1},K}$	$m_{c_{2},K}$	$m_{c_{1},Q}$	$\mu_{B}$	$\mu_{SF}$
0.32	0.40	0.29	0.51	0.15	0.18

## Data Availability

The data supporting this study’s findings are available from the corresponding author upon reasonable request.

## Supplementary Materials

The following supporting information can be downloaded at: https://www.mdpi.com/article/10.3390/nano13111739/s1, theory for the non-local dielectric function of layered structure; summary of the dielectric constants of capping and substrate materials (Table S1); summary of the measured BX-DX splittings of WSe${}_{2}$ monolayers in various dielectric environments reported in the literature (Table S2); the extended hydrogen model of exciton with non-local dielectric screening;convergence test of DFT calculation (Figures S2–S4); the validity of Wannier tight-binding model (Figure S5 and Table S3) and the determination of effective masses (Figure S6 and Table S4). References [73,74,75,76,77,78,79,80,81,82,83] are cited in the supplementary materials.
