# Supplementary material for: The Key Role of Non-Local Screening in the Environment-Insensitive Exciton Fine Structures of Transition-Metal Dichalcogenide Monolayers
Source: Nanomaterials (Basel). 2023 May 26;13(11):1739. doi: 10.3390/nano13111739 (PMC10254311; doi:10.3390/nano13111739)
Supplement: Supplementary file 1 [file nanomaterials-13-01739-s001.zip › nanomaterials-2370993-supplementary.pdf]

Supporting Information: The key role of non-local screening in the environment-insensitive exciton fine structures of transition-metal dichalcogenide monolayers

Wei-Hua Li,† Jhen-Dong Lin,† Ping-Yuan Lo,† Guan-Hao Peng,† Ching-Yu Hei,† Shao-Yu Chen,‡ and Shun-Jen Cheng*,†

†*Department of Electrophysics, National Yang Ming Chiao Tung University, Hsinchu 300, Taiwan*

‡*Center of Condensed Matter Sciences, National Taiwan University, Taipei 106, Taiwan*

¶*Center of Atomic Initiative for New Material, National Taiwan University, Taipei 106, Taiwan*

E-mail: sjcheng@nycu.edu.tw

Non-local dielectric function of layered structure

The matrix elements of the electron-hole (e-h) direct Coulomb interaction in the Bethe-Salpeter equation (BSE) represent the amplitudes of the Coulomb scattering between the charge pair densities via the screened Coulomb interaction. The screened Coulomb interaction is essentially the Green's function that describes the interaction between two point charges. With the dielectric screening of media, the screened Coulomb interaction W at $\boldsymbol{r}_1$

with a point charge located $\boldsymbol{r}_2$ obeys the Poisson's equation[1–4]

$$\nabla_{\boldsymbol{r}_1} \cdot \int d^3\boldsymbol{r}' \, \varepsilon(\boldsymbol{r}_1, \boldsymbol{r}') \nabla_{\boldsymbol{r}'} W(\boldsymbol{r}', \boldsymbol{r}_2) = -\frac{e^2}{\varepsilon_0} \delta(\boldsymbol{r}_1 - \boldsymbol{r}_2), \tag{S1}$$

where $\varepsilon(\boldsymbol{r}_1, \boldsymbol{r}')$ is the dielectric function. Due to the lattice translational symmetries of the screened Coulomb interaction and the dielectric function in 2D plane, we can expand the screened Coulomb interaction and the dielectric function by the in-plane Fourier series which is defined as

$$f_{\boldsymbol{G}_1\boldsymbol{G}_2}(\boldsymbol{q}; z_1, z_2) = \frac{1}{\mathcal{A}} \int d^2\boldsymbol{\rho}_1 d^2\boldsymbol{\rho}_2 \, e^{-i(\boldsymbol{q}+\boldsymbol{G}_1)\cdot\boldsymbol{\rho}_1} f(\boldsymbol{\rho}_1, z_1, \boldsymbol{\rho}_2, z_2) e^{i(\boldsymbol{q}+\boldsymbol{G}_2)\cdot\boldsymbol{\rho}_2}, \tag{S2}$$

where $\boldsymbol{G}$ is the reciprocal lattice vector and $\boldsymbol{\rho} = (x, y)$ is the in-plane coordinate. By using the Fourier series, the Poisson's equation is reformulated as

$$\sum_{\boldsymbol{G}'} \int dz' \left[-(\boldsymbol{q}+\boldsymbol{G}_1)\cdot(\boldsymbol{q}+\boldsymbol{G}')\varepsilon_{\boldsymbol{G}_1\boldsymbol{G}'}(\boldsymbol{q}; z_1, z') + \frac{\partial}{\partial z_1}\varepsilon_{\boldsymbol{G}_1\boldsymbol{G}'}(\boldsymbol{q}; z_1, z')\frac{\partial}{\partial z'} \right] W_{\boldsymbol{G}'\boldsymbol{G}_2}(\boldsymbol{q}; z', z_2) = -\frac{e^2}{\varepsilon_0}\delta_{\boldsymbol{G}_1\boldsymbol{G}_2}\delta(z_1 - z_2). \tag{S3}$$

Since the e-h direct Coulomb interaction is dominated by the long-range component,[1,5,6] we take the long-range approximation ($\boldsymbol{G}_1 = \boldsymbol{G}_2 = \boldsymbol{G}' = \boldsymbol{0}$) for the Poisson's equation Eq. (S3), and assume that the dielectric function is local in z-direction,[1–3]

$$\varepsilon_{\boldsymbol{00}}(\boldsymbol{q}; z_1, z') \approx \varepsilon_{\boldsymbol{00}}(\boldsymbol{q}; z_1)\delta(z_1 - z'). \tag{S4}$$

For simplification of the notations, hereafter we omit the indices of reciprocal lattice vector $\boldsymbol{G}$, and the notations are changed as $\varepsilon_{\boldsymbol{q}}(z_1) = \varepsilon_{\boldsymbol{00}}(\boldsymbol{q}; z_1)$ and $W(\boldsymbol{q}; z_1, z_2) = W_{\boldsymbol{00}}(\boldsymbol{q}; z_1, z_2)$. Accordingly, the screened Coulomb interaction in the momentum space can be obtained by

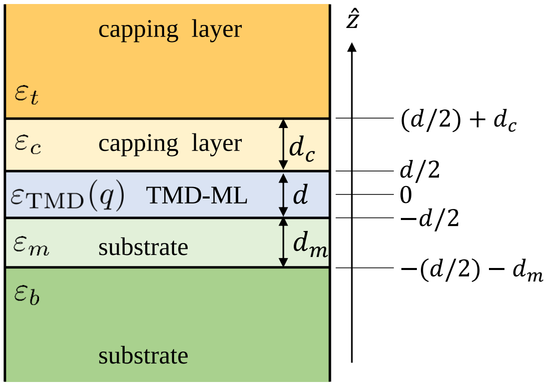

Figure S1: The schematics of the five-layer structure with TMD embedded in the middle, which gives rise to the piece-wise dielectric function of Eq. (S6). The q-dependent bulk dielectric function of TMD, $\varepsilon_{\mathrm{TMD}}(q)$, is adopted for the layer of TMD-ML. ε_b, ε_m, ε_c and ε_t are the dielectric constants of the environmental media. d_m and d_c are the thicknesses of the middle substrate and the middle capping layer, respectively.

solving the Poisson equation

$$\left[-q^2\varepsilon_{\boldsymbol{q}}(z_1)+\frac{\partial}{\partial z_1}\varepsilon_{\boldsymbol{q}}(z_1)\frac{\partial}{\partial z_1}\right]W(\boldsymbol{q};z_1,z_2)=-\frac{e^2}{\varepsilon_0}\delta(z_1-z_2). \tag{S5}$$

Following the approach of Refs.[1–3], the dielectric function is assumed to be isotropic in 2D plane and is taken as the piece-wise function in z-direction with the values depending on the positions of TMD-ML, substrates and capping layers (see Fig. S1). For the 5-layer structure, $\varepsilon_{\boldsymbol{q}}(z_1)$ is given by

$$\varepsilon_{\boldsymbol{q}}(z_1)=\begin{cases}\varepsilon_t, & z_1>\frac{d}{2}+d_c\\ \varepsilon_c, & \frac{d}{2}<z_1<\frac{d}{2}+d_c\\ \varepsilon_{\mathrm{TMD}}(q), & -\frac{d}{2}<z_1<\frac{d}{2}\\ \varepsilon_m, & -\frac{d}{2}-d_m<z_1<-\frac{d}{2}\\ \varepsilon_b, & z_1<-\frac{d}{2}-d_m\end{cases} \tag{S6}$$

where $q=|\boldsymbol{q}|$, ε_t, ε_c, ε_m and ε_b are the dielectric constants of layers surrounding TMD-ML,

Table S1: Dielectric constants of environmental materials in this work.

Material	SiO_2 [9]	hBN [10,11]	Al_2O_3 [12]	HfO_x [13]
Dielectric constant	3.9	4.5	9.2	15

d is the thickness of TMD-ML layer, d_c and d_m are the thickness of the middle capping layer and middle substrate with dielectric constant ε_c and ε_m, respectively. For the layer of TMD-ML, we adopt the q-dependent dielectric function of bulk TMD in the random-phase approximation [7,8]

$$\varepsilon_{\text{TMD}}(q) = 1 + \frac{1}{(\varepsilon_{2D} - 1)^{-1} + \alpha \frac{q^2}{q_{TF}^2} + (\frac{\hbar^2 q^2}{2m_0 E_{pl}})^2} \tag{S7}$$

where ε_{2D} is the in-plane dielectric constant of the bulk TMD, E_{pl} is the plasma peak energy, $\alpha = 1.55$ [8] is the fitting parameter and $q_{TF} = \sqrt{\frac{e^2 m_0}{\pi^2 \varepsilon_0 \hbar^2}(\frac{3\pi^2 \varepsilon_0 m_0 E_{pl}^2}{e^2 \hbar^2})^{1/3}}$ is the Thomas-Fermi wave vector. [1,8]

Based on the piece-wise dielectric function in z-direction and the vanished potential at infinities ($z \to \pm\infty$), the solution of the screened Coulomb interaction with the source charge located in the layer of TMD-ML, i.e., $-\frac{d}{2} < z_2 < \frac{d}{2}$, has the form

$$W(\boldsymbol{q}, z_1, z_2) = \frac{e^2}{2q\varepsilon_0 \varepsilon_{\boldsymbol{q}}(z_1)} \begin{cases} C_1 e^{-qz_1}, & z_1 > \frac{d}{2} + d_c \\ C_2 e^{-qz_1} + C_3 e^{qz_1}, & \frac{d}{2} < z_1 < \frac{d}{2} + d_c \\ C_4 e^{-qz_1} + C_5 e^{qz_1} + e^{-q|z_1 - z_2|}, & -\frac{d}{2} < z_1 < \frac{d}{2} \\ C_6 e^{-qz_1} + C_7 e^{qz_1}, & -\frac{d}{2} - d_m < z_1 < -\frac{d}{2} \\ C_8 e^{qz_1}, & z_1 < -\frac{d}{2} - d_m \end{cases} \tag{S8}$$

The coefficients C_i can be found out by the electrostatic boundary conditions [1,3,14]

$$W(\boldsymbol{q}; z_1 = d_i^+, z_2) = W(\boldsymbol{q}; z_1 = d_i^-, z_2) \tag{S9}$$

$$\varepsilon_{\boldsymbol{q}}(z_1 = d_i^+) \frac{\partial}{\partial z_1} W(\boldsymbol{q}; z_1, z_2)\Big|_{z_1 = d_i^+} = \varepsilon_{\boldsymbol{q}}(z_1 = d_i^-) \frac{\partial}{\partial z_1} W(\boldsymbol{q}; z_1, z_2)\Big|_{z_1 = d_i^-} \tag{S10}$$

where d_i denotes the the positions of the interfaces in the z-coordinate. After deriving $W(\boldsymbol{q}; z_1, z_2)$, the effective dielectric function is defined as the ratio of the z-averaged unscreened Coulomb interaction to the screened one

$$\varepsilon(\boldsymbol{q}) = V(\boldsymbol{q})/W(\boldsymbol{q}), \tag{S11}$$

where

$$W(\boldsymbol{q}) = \frac{1}{d^2}\int_{-d/2}^{d/2}\int_{-d/2}^{d/2} dz_1 dz_2\, W(\boldsymbol{q}, z_1, z_2), \tag{S12}$$

and

$$\begin{aligned} V\left(\boldsymbol{q}\right) =& \frac{1}{d^2}\int_{-d/2}^{d/2} dz_1 \int_{-d/2}^{d/2} dz_2 \int d^2\boldsymbol{\rho}\, V\left(\boldsymbol{\rho}; z_1 - z_2\right) e^{-i\boldsymbol{q}\cdot\boldsymbol{\rho}} \\ =& \frac{e^2}{4\pi\varepsilon_0}\frac{4\pi}{dq^2}\left(1 - \frac{1-e^{-qd}}{qd}\right). \end{aligned} \tag{S13}$$

The dielectric function of the 5-layer structure is given by

$$\varepsilon(\boldsymbol{q}) = \frac{\varepsilon_{\rm TMD}(q)}{D(\boldsymbol{q})} \tag{S14}$$

where

$$
\begin{aligned}
D(\boldsymbol{q}) =& 1 + \Bigg\{ \bigg\{ e^{-2dq}(e^{dq}-1)^2 \\
& \times \Big[\sinh(d_m q)\Big(-e^{dq}\big(\sinh(d_c q)(\varepsilon_m^2\varepsilon_c^2 - \varepsilon_{\text{TMD}}^2(q)\varepsilon_b\varepsilon_t) - \varepsilon_c \cosh(d_c q)(\varepsilon_{\text{TMD}}^2(q)\varepsilon_b - \varepsilon_t\varepsilon_m^2)\big) \\
& + (\varepsilon_{\text{TMD}}(q)\varepsilon_b - \varepsilon_m^2)\big(\sinh(d_c q)(\varepsilon_{\text{TMD}}(q)\varepsilon_t - \varepsilon_c^2) \\
& + \cosh(d_c q)\varepsilon_c(\varepsilon_{\text{TMD}}(q) - \varepsilon_t)\big)\Big) \\
& + \varepsilon_m \cosh(d_m q)\Big(e^{dq}\big(\sinh(d_c q)(\varepsilon_{\text{TMD}}^2(q)\varepsilon_t - \varepsilon_b\varepsilon_c^2) - \varepsilon_c \cosh(d_c q)(\varepsilon_b\varepsilon_t - \varepsilon_{\text{TMD}}^2(q))\big) \\
& + (\varepsilon_{\text{TMD}}(q) - \varepsilon_b)\big(\sinh(d_c q)(\varepsilon_{\text{TMD}}(q)\varepsilon_t - \varepsilon_c^2) \\
& + \varepsilon_c \cosh(d_c q)(\varepsilon_{\text{TMD}}(q) - \varepsilon_t)\big)\Big)\Big]\bigg\} \\
& \times \bigg\{ 2\sinh(dq)\Big[\sinh(d_m q)\Big(\sinh(d_c q)\big(\varepsilon_m^2\varepsilon_c^2 + \varepsilon_{\text{TMD}}^2(q)\varepsilon_b\varepsilon_t\big) + \varepsilon_c \cosh(d_c q)(\varepsilon_{\text{TMD}}^2(q)\varepsilon_b + \varepsilon_t\varepsilon_m^2)\Big) \\
& + \varepsilon_m \cosh(d_m q)\Big(\sinh(d_c q)\big(\varepsilon_{\text{TMD}}^2(q)\varepsilon_t + \varepsilon_b\varepsilon_c^2\big) \\
& + \varepsilon_c \cosh(d_c q)\big(\varepsilon_b\varepsilon_t + \varepsilon_{\text{TMD}}^2(q)\big)\Big)\Big] \\
& + 2\varepsilon_{\text{TMD}}(q)\cosh(dq)\Big[\sinh(d_m q)\Big(\sinh(d_c q)\big(\varepsilon_t\varepsilon_m^2 + \varepsilon_b\varepsilon_c^2\big) + \varepsilon_c \cosh(d_c q)\big(\varepsilon_b\varepsilon_t + \varepsilon_m^2\big)\Big) \\
& + \varepsilon_m \cosh(d_m q)\Big(\sinh(d_c q)\big(\varepsilon_b\varepsilon_t + \varepsilon_c^2\big) + \varepsilon_c \cosh(d_c q)\big(\varepsilon_b + \varepsilon_t\big)\Big)\Big]\bigg\}^{-1} \\
& \times (e^{-dq} + dq - 1)^{-1} \Bigg\}.
\end{aligned} \tag{S15}
$$

For the parameters in the bulk dielectric function $\varepsilon_{\text{TMD}}(q)$ of WSe_2-ML, we adopt $\alpha = 1.55$,[8] the thickness $d = 6.72$ Å,[15] the in-plane dielectric constant $\varepsilon_{2D} = 13.8$,[16] and the plasma peak energy $E_{pl} = 22.6$ eV.[17] The used dielectric constants of capping layers and substrates in this work are listed in Table. S1.

After deriving the dielectric function and the screened Coulomb interaction of TMD-ML, the matrix elements of the *e*-*h* direct Coulomb interaction in the Bethe-Salpeter equation can be calculated. The methodology of calculating the matrix elements of the electron-hole Coulomb interaction are detailed in the supporting information of our previous publication

Table S2: Summary of the measured fine structure splittings between BX and SFDX states of WSe_2 monolayers embedded in different dielectric structures by the existing experiments. The superscript $*$ marks the calculated splittings by this work.

Dielectric structure	Measured $\Delta^{X}_{B,SF}$ (meV)	References
Air/WSe_2/SiO_2	51.2*	Our BSE calculation
Air/WSe_2/SiO_2	47	[18]
Air/WSe_2/SiO_2	47	[19]
Air/WSe_2/hBN	49.9*	Our BSE calculation
Air/WSe_2/hBN	43	[20]
hBN/WSe_2/SiO_2	43.7*	Our BSE calculation
hBN/WSe_2/SiO_2	46	[20]
hBN/WSe_2/hBN	42.7*	Our BSE calculation
hBN/WSe_2/hBN	41.8	Our experiment work
hBN/WSe_2/hBN	38	[21]
hBN/WSe_2/hBN	40	[22]
hBN/WSe_2/hBN	40	[23–25]
hBN/WSe_2/hBN	41	[26–28]
hBN/WSe_2/hBN	41	[29]
hBN/WSe_2/hBN	42	[30]
hBN/WSe_2/hBN	42	[31]
hBN/WSe_2/hBN	42	[32]
hBN/WSe_2/hBN	43	[33]
hBN/WSe_2/hBN	43	[34]
hBN/WSe_2/hBN	43	[35]
hBN/WSe_2/hBN	43	[36,37]
hBN/WSe_2/hBN	44	[20]

Ref.[5].

Comparison of the BSE theoretical simulation and experimental observations in the literature

We make the comparison of the BSE-calculated and experimentally measured BX-SFDX splittings of WSe_2-ML in the dielectric structures of air/WSe_2/SiO_2, air/WSe_2/hBN, hBN/WSe_2/SiO_2 and hBN/WSe_2/hBN, as summarized by Table. S2.,[18–37] Basically, the BSE-calculated splittings are in fairly good agreement with the measured ones. From the data of Table. S2, one can note that the magnitudes of the fine structure splittings of WSe_2-ML in the different

dielectric structures are weakly environment-dependent.

The effective dielectric constant

In the extended hydrogen model, we estimate the effective dielectric constant of S-state exciton by averaging the dielectric function in $\boldsymbol{q}$-space within the circle $|\boldsymbol{q}| \leq 2/a_S^X$ [38]

$$\varepsilon_{eff,S} \approx 2\left(\frac{a_S^X}{2}\right)^2 \int_0^{2/a_S^X} dq\, q\varepsilon(q) \tag{S16}$$

where $a_S^X = \frac{4\pi\varepsilon_0\hbar^2}{e^2}\frac{\varepsilon_{eff,S}}{\mu_S}$ is the exciton Bohr radius, and μ_S is the reduced mass of the exciton. Eq. (S16) is essentially a self-consistent equation.

For simplicity, we consider the cases of the the TMD-ML encapsulated by two semi-infinite dielectrics by employing $\varepsilon_t = \varepsilon_c = \varepsilon_m = \varepsilon_b = \varepsilon_{env}$ in the model calculations for Fig.4 of the main article. In addition, because the dielectric function of bulk TMD weakly depends on q when q is small, we approximate $\varepsilon_{\mathrm{TMD}}(q) \approx \varepsilon_{2D} = 13.8$ for WSe_2. By expanding $\varepsilon(q)$ in the Taylor series with respect to q up to the first- or second-order terms, $\varepsilon_{eff,S}$ is solvable according to Eq. (S16).

First-order approximation

In the first-order approximation, we take $\varepsilon(q) \approx c_0 + c_1 q$ and, from Eq. (S16), derive

$$\varepsilon_{eff,S} = c_0 + \frac{e^2}{3\pi\varepsilon_0\hbar^2}\frac{\mu_S}{\varepsilon_{eff,S}}c_1\,. \tag{S17}$$

Accordingly, we solve

$$\varepsilon_{eff,S} \approx \varepsilon_{eff,S}^{(1)} = \frac{c_0}{2} + \frac{1}{2}\sqrt{c_0^2 + \frac{4e^2}{3\pi\varepsilon_0\hbar^2}\mu_S c_1}\,. \tag{S18}$$

Defining $\Delta\varepsilon = \varepsilon_{eff,D} - \varepsilon_{eff,B}$, one can show

$$\Delta\varepsilon = \frac{1}{2}\sqrt{c_0^2 + \frac{4e^2 c_1}{3\pi\varepsilon_0\hbar^2}\mu_D} - \frac{1}{2}\sqrt{c_0^2 + \frac{4e^2 c_1}{3\pi\varepsilon_0\hbar^2}\mu_B}\,. \tag{S19}$$

in the first-order approximation for the Taylor-expanded $\varepsilon(q)$ with respect to q.

Second-order approximation

For the better quantitative evaluation of $\varepsilon_{eff,S}$, one can take the second-order approximation of $\varepsilon(q)$ to solve Eq. (S16), which gives rise to

$$\varepsilon_{eff,S} = c_0 + \frac{e^2}{3\pi\varepsilon_0\hbar^2}\frac{\mu_S}{\varepsilon_{eff,S}}c_1 + 2\left(\frac{e^2}{4\pi\varepsilon_0\hbar^2}\right)^2\frac{\mu_S^2}{\varepsilon_{eff,S}^2}c_2. \tag{S20}$$

Because the range of the integration of Eq. (S16) is not far from $q = 0$, the magnitude of the third term in RHS of Eq. (S20), which comes from the q^2-term of $\varepsilon(q)$, is small. To avoid the complicate solution of the cubic equation, instead, we treat the third term in RHS of Eq. (S20) as a small correction factor and approximate the $\varepsilon_{eff,S}$ in RHS of Eq. (S20) as the solution derived from the first-order approximation. (If one ignored the last term of Eq. (S20), the solution of the second-order approximation would just return to that of the first-order approximation.) Therefore, the solution of the second-order approximation is given by

$$\begin{aligned}\varepsilon_{eff,S} &\approx \varepsilon_{eff,S}^{(1)} + 2\left(\frac{e^2}{4\pi\varepsilon_0\hbar^2}\right)^2\frac{\mu_S^2 c_2}{\left(\varepsilon_{eff,S}^{(1)}\right)^2} \\ &= \frac{c_0}{2} + \frac{1}{2}\sqrt{c_0^2 + \frac{4e^2}{3\pi\varepsilon_0\hbar^2}\mu_S c_1} + 2\left(\frac{e^2}{4\pi\varepsilon_0\hbar^2}\right)^2\frac{\mu_S^2 c_2}{\left(\frac{c_0}{2} + \frac{1}{2}\sqrt{c_0^2 + \frac{4e^2}{3\pi\varepsilon_0\hbar^2}\mu_S c_1}\right)^2}.\end{aligned} \tag{S21}$$

Extended 2D-hydrogen model with the non-local dielectric screening

The energy splitting between the bright exciton (BX) and dark excitons (DX) is determined by

$$\Delta^{X}_{B,D} = \Delta_c + \Delta^{X(d)}_{B,D} + \Delta^{X(xc)}_{B,D}, \tag{S22}$$

where the conduction band splitting $\Delta_c = 19.6$ meV from the DFT calculation, $\Delta^{X(d)}_{B,D}$ is the direct-interaction-induced binding energy difference between BX and DX and $\Delta^{X(xc)}_{B,D}$ is the difference of the exchange energy between BX and DX. According to the discussion of the previous section, DX experiences the larger effective dielectric constant due to its heavier mass, i.e., $\varepsilon_{eff,D} = \varepsilon_{eff} + \Delta\varepsilon$, where $\varepsilon_{eff} \equiv \varepsilon_{eff,B}$. In the 2D-hydrogen model, the binding energy difference of BX and DX is given by

$$\begin{aligned}\Delta^{X(d)}_{B,D} &= E^b_D - E^b_B \\ &= 4Ry\left[\frac{\mu_D/m_0}{(\varepsilon_{eff}+\Delta\varepsilon)^2} + \frac{\mu_B/m_0}{\varepsilon^2_{eff}}\right] \\ &\approx 4Ry\left[\frac{\Delta\mu_{DB}/m_0}{\varepsilon^2_{eff}} - 2\Delta\varepsilon\frac{\mu_D/m_0}{\varepsilon^3_{eff}}\right],\end{aligned} \tag{S23}$$

where $\Delta\mu_{DB} = \mu_D - \mu_B$ is the difference of the reduced mass between BX and DX. In Eq. (S23), we take the first-order term of $\Delta\varepsilon$ to carry out the influence of the $\Delta\varepsilon$ on the BX-DX splitting.

The third term in Eq. (S26) is evaluated by $\Delta^{X}_{B,D} = V^x_B - V^x_D$, where V^x_S is the exchange energy of exciton state S. For the dipole-forbidden SFDX, $V^x_D = 0$ is vanishing. Hence, $\Delta^{X(xc)}_{B,D}$ is contributed solely from the exchange energy of BX. The exchange energy of an exciton state S is evaluated by

$$V^x_S = \frac{1}{\mathcal{A}}\sum_{vc\boldsymbol{k}}\sum_{v'c'\boldsymbol{k}'} A^{(0)*}_{S,\boldsymbol{k}_{ex}}(vc\boldsymbol{k}) V^x_{\boldsymbol{k}_{ex}}(vc\boldsymbol{k}, v'c'\boldsymbol{k}') A^{(0)}_{S,\boldsymbol{k}_{ex}}(v'c'\boldsymbol{k}'), \tag{S24}$$

where $A^{(0)}_{S,\boldsymbol{k}_{ex}}(vc\boldsymbol{k})$ is the amplitude of the e-h configuration of the exchange-free exciton state, $V^x_{\boldsymbol{k}_{ex}}(vc\boldsymbol{k}, v'c'\boldsymbol{k}')$ is the kernel of the e-h exchange Coulomb interaction, and $\mathcal{A}$ is the area of TMD-ML. Similar to the way to evaluate the effective dielectric constant in Eq. (S16), we consider the exciton wavefunction $A^{(0)}_{S,\boldsymbol{k}_{ex}}(vc\boldsymbol{k})$ to be localized around the K or K' valley and simplified to be a constant as $|\boldsymbol{k} - \boldsymbol{K}| \leq 2/a^X_S$. Under the simplification, the difference of exchange energies between BX and DX can be estimated by

$$\Delta^{X(xc)}_{B,D} \approx \frac{(\mu_B/m_0)^2}{\pi(a_0)^2} \frac{1}{\varepsilon^2_{eff}} \bar{v}^x(\boldsymbol{q}_0), \tag{S25}$$

where $a_0 = 0.53$ Å is the Bohr radius in the hydrogen atom, $\bar{v}^x(\boldsymbol{q}_0) \equiv \mathcal{A}V^x_{\boldsymbol{0}}(vc\boldsymbol{K}+\boldsymbol{q}_0, vc\boldsymbol{K}+\boldsymbol{q}_0)$ and $\boldsymbol{q}_0$ are the characteristic wave vectors for a BX with the magnitude $q_0 = 1/a^X_B$.

In Eq. (S23), the second term with $\Delta\varepsilon$ arises from the non-local dielectric screening and makes a negative ε^{-3}_{eff}-contribution to the BX-DX splitting. Thus, one can rewrite Eq. (S22) in terms of the parts respectively arising from the local and non-local screenings

$$\Delta^X_{B,D} = \Delta^{X(local)}_{B,D} + \Delta^{X(non-loc.)}_{B,D}, \tag{S26}$$

where the part contributed from the effective local screening is defined as

$$\Delta^{X(local)}_{B,D} = \Delta_c + 4\varepsilon^{-2}_{eff}\left[(\Delta\mu_{DB}/m_0)Ry + \frac{(\mu_B/m_0)^2}{4\pi(a_0)^2}\bar{v}^x(\boldsymbol{q}_0)\right], \tag{S27}$$

and the part contributed from the non-local screening is defined as

$$\Delta^{X(non-loc.)}_{B,D} = -8\varepsilon^{-3}_{eff}\Delta\varepsilon(\mu_D/m_0)Ry. \tag{S28}$$

Eqs. (S26) - (S28) constitute the extended exciton model with the consideration of non-local screening used for Fig.4 in the main article.

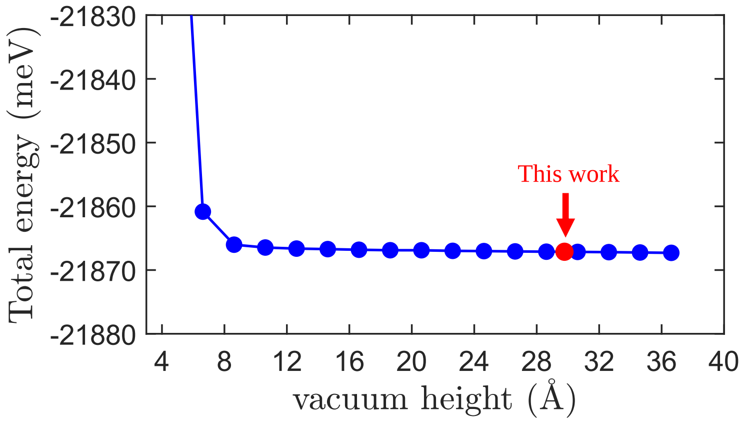

Figure S2: Convergence of total energy of the system with respect to the vacuum height in the DFT calculations with the use of the PBE exchange-correlation functional and the fixed cut-off energy 500 eV and the $9 \times 9 \times 1$ k-grids. The vacuum height 29.78 Å used for the simulations in the main article is marked by the red circle.

Convergence test of DFT calculation

Since the conduction band splitting $\Delta_c \approx 20$ meV is crucial to the exciton fine structure splitting of WSe_2-ML, we perform the convergences tests with respect to the cut-off energy, vacuum height and density of k-grids to verify the validity of the DFT calculations. Firstly, we perform the convergence of the total energy of the system with respect to the vacuum height in the exchange-correlation functional of Perdew-Burke-Ernzerhof (PBE)[39] generalized gradient approximation. As shown by Figure S2, with increasing the vacuum height from 4.4 Å to 36.6 Å, the total energy varies between -21.87 eV and -21.86 eV and quickly converges to -21.872 eV as the vacuum height exceeds 10.6 Å. The difference between the total energies of the system at vacuum height 10.6 Å and 36.6 Å is only 0.8 meV. This justifies that the vacuum height 29.78 Å used in our work is sufficiently long to guarantee the convergence of the total energy.

Next, we perform the tests of convergences of the total energy and the conduction band splitting Δ_c with respect to the cut-off energy with the use of the HSE06 exchange-correlation functional. As shown by Figure S3, the variation of the total energy of the system is less than 1 meV and that of Δ_c is 0.1 meV with varying the cut-off energy from $400 \sim 700$ eV.

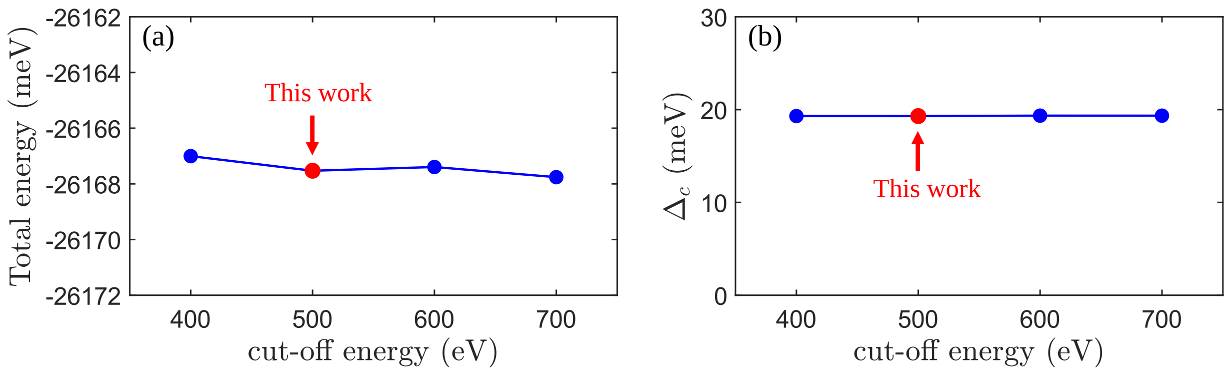

Figure S3: Convergences of (a) the total energy of the system and (b) the conduction band splitting with respect to the cut-off energy in the DFT calculations with the use of the HSE06 exchange-correlation functional. The vacuum height is fixed at 29.78 Å and k-grid is fixed at $9 \times 9 \times 1$ for the convergence test with varied cut-off energy. The cut-off energy 500 eV used for the simulations presented in the main article are marked by the red circles.

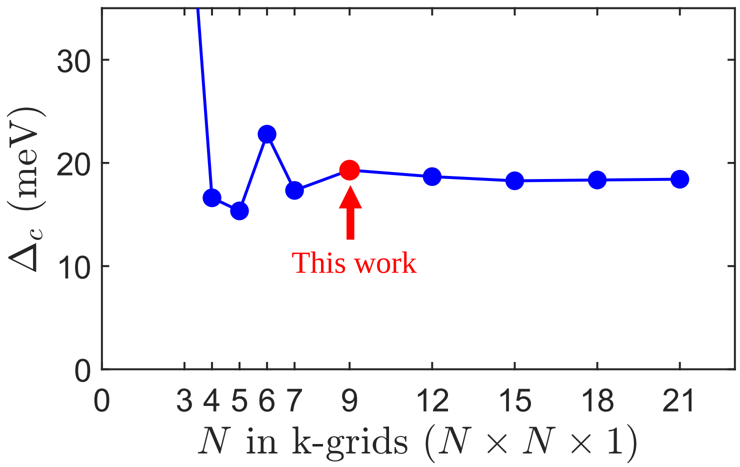

Figure S4: Convergence of the conduction band splitting energy with respect to the number of k-grids in DFT calculations with the use of the HSE06 exchange-correlation functional. The vacuum height is fixed at 29.78 Å and the cut-off energy is fixed at 500 eV. The k-grids $9 \times 9 \times 1$ used for the simulations presented in the main article is marked by the red circle.

Accordingly, the both total energy and Δ_c are justified to well converge as the cut-off energy is set to be 500 eV.

Based on the verified vacuum height and cut-off energy, we perform the convergence of Δ_c with respect to the number of k-grids in the DFT-HSE06 calculations, which is shown in Figure S4. With increasing the number of k-girds from $3 \times 3 \times 1$ to $21 \times 21 \times 1$, the calculated conduction band splitting is nearly unchanged and converge to the value of $\Delta_c \approx 20$ meV as

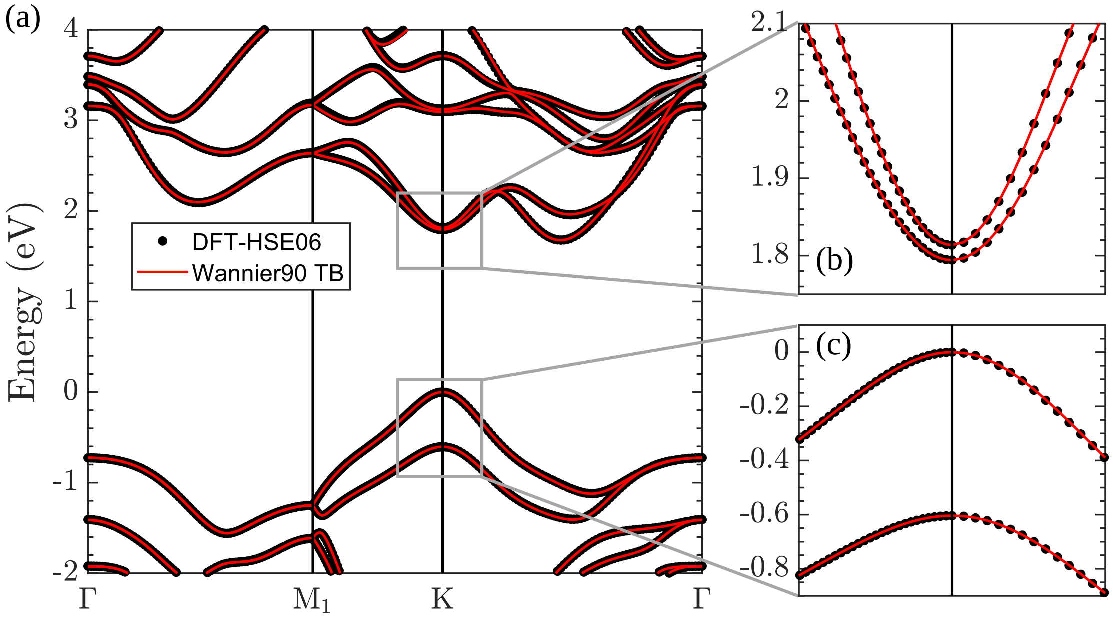

Figure S5: (a) Comparison of the band structures of WSe_2-ML calculated by DFT (black sphere) and Wannier90 TB (red line). (b) [(c)] The close-up of the conduction [valence] bands around K valley.

Table S3: The conduction band splitting Δ_c and the valence band splitting Δ_v at K point in DFT-HSE06 and Wannier90 TB model.

	DFT-HSE06	Wannier90 TB	Difference
Δ_c	19.303 meV	19.635 meV	0.332 meV
Δ_v	604.066 meV	603.811 meV	0.255 meV

the number of k-grids exceeds $9 \times 9 \times 1$, which is taken in our DFT calculations throughout this work. To save the computational cost, we choose the number of k-grids $9 \times 9 \times 1$ in the DFT calculation for the studies of the exciton fine structure splittings throughout this work.

The validity of Wannier tight-binding model

Figure S5 presents the comparison of the band structures of WSe_2-ML calculated by DFT-HSE06 and Wannier90 tight-binding (TB) model. Overall, the calculated energy dispersions by Wannier90 TB model are in a highly agreement with those by DFT. Additionally, Table S3 shows the energy splittings of conduction and valence bands at K point in DFT-HSE06 and Wannier90 TB model. The conduction band splitting Δ_c calculated by using DFT and

Table S4: The MAEs in the least square fitting of the effective masses for the conduction and valence band edges at the high symmetry points $\boldsymbol{k}_0 = K$ and Q

	$m_{v_1,K}$	$m_{c_1,K}$	$m_{c_2,K}$	$m_{c_1,Q}$
mean absolute error (MAE)	0.56 meV	0.44 meV	1.04 meV	0.84 meV

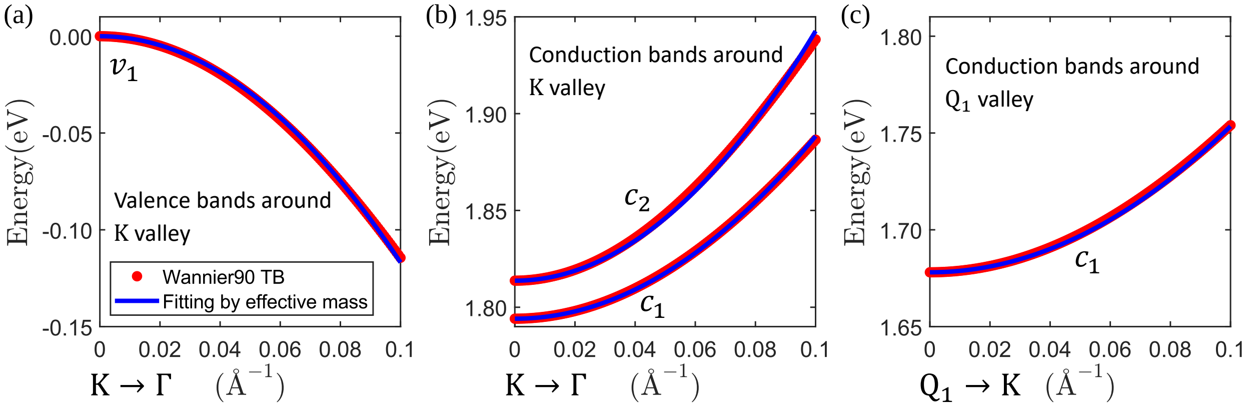

Figure S6: Comparisons of the DFT-calculated band dispersions and the ideally parabolic dispersions with the fitted effective masses of (a) valence bands around K valley, (b) conduction bands around K valley and (c) conduction bands around Q_1 valley.

Wannier TB are 19.303 meV and 19.635 meV, respectively, and differ by 0.332 meV, which is negligible as compared with the magnitude of Δ_c.

Determination of the effective masses

The effective mass of the n-th quasi-particle band dispersion is obtained by means of least square fitting, which fits the parabolic band, $\widetilde{\epsilon}_{n,\boldsymbol{k}} \approx \epsilon_{n,\boldsymbol{k}_0} + \frac{\hbar^2|\boldsymbol{k}-\boldsymbol{k}_0|^2}{2m_{n,\boldsymbol{k}_0}}$, with well-defined effective mass $m_{n,\boldsymbol{k}_0}$ to the DFT-calculated band dispersion over the k-range $0 < |\boldsymbol{k} - \boldsymbol{k}_0| < 0.1\text{Å}^{-1}$ with the least mean absolute error (MAE). The k-range over which the least square fitting is carried out is determined according to the extent of the k-space wavefunction of the exciton. The mean absolute error measures the average error of each fitting point and is defined by $\text{MAE} = \frac{1}{N}\sum_{i=1}^{N} |\widetilde{\epsilon}_{n,\boldsymbol{k}_i} - \epsilon_{n,\boldsymbol{k}_i}|$, where $N = 150$ is the number of k-grids. The MAEs in the least square fitting of the effective masses for the conduction and valence band edges at the high symmetry points $\boldsymbol{k}_0 = K$ and Q, are summarized in the Table S4. In all the cases, the MAEs is less than or comparable to one meV only, which is extremely small as compared

with the energy scale of the band dispersion over the range $0 < |\boldsymbol{k} - \boldsymbol{k}_0| < 0.1\text{Å}^{-1}$ which is about 100 meV (See Figure S6). The comparisons of the DFT-calculated band dispersions with the parabolic bands with the fitted effective masses by the least square method are presented in the Figure S6, showing an excellent agreement.

References

(1) Trolle, M. L.; Pedersen, T. G.; Véniard, V. Model dielectric function for 2D semiconductors including substrate screening. *Scientific reports* **2017**, *7*, 39844.

(2) Florian, M.; Hartmann, M.; Steinhoff, A.; Klein, J.; Holleitner, A. W.; Finley, J. J.; Wehling, T. O.; Kaniber, M.; Gies, C. The Dielectric Impact of Layer Distances on Exciton and Trion Binding Energies in van der Waals Heterostructures. *Nano Letters* **2018**, *18*, 2725–2732, PMID: 29558797.

(3) Kamban, H. C.; Pedersen, T. G.; Peres, N. M. R. Anisotropic Stark shift, field-induced dissociation, and electroabsorption of excitons in phosphorene. *Phys. Rev. B* **2020**, *102*, 115305.

(4) Kornyshev, A. A.; Rubinshtein, A. I.; Vorotyntsev, M. A. Model nonlocal electrostatics. I. *Journal of Physics C: Solid State Physics* **1978**, *11*, 3307.

(5) Peng, G.-H.; Lo, P.-Y.; Li, W.-H.; Huang, Y.-C.; Chen, Y.-H.; Lee, C.-H.; Yang, C.-K.; Cheng, S.-J. Distinctive Signatures of the Spin- and Momentum-Forbidden Dark Exciton States in the Photoluminescence of Strained WSe2 Monolayers under Thermalization. *Nano Letters* **2019**, *19*, 2299–2312, PMID: 30860847.

(6) Latini, S.; Olsen, T.; Thygesen, K. S. Excitons in van der Waals heterostructures: The important role of dielectric screening. *Phys. Rev. B* **2015**, *92*, 245123.

(7) Cappellini, G.; Del Sole, R.; Reining, L.; Bechstedt, F. Model dielectric function for semiconductors. *Phys. Rev. B* **1993**, *47*, 9892–9895.

(8) Katsch, F.; Selig, M.; Knorr, A. Exciton-Scattering-Induced Dephasing in Two-Dimensional Semiconductors. *Phys. Rev. Lett.* **2020**, *124*, 257402.

(9) Neamen, D. A. *Semiconductor physics and devices: basic principles*; McGraw-hill, 2012.

(10) Geick, R.; Perry, C. H.; Rupprecht, G. Normal Modes in Hexagonal Boron Nitride. *Phys. Rev.* **1966**, *146*, 543–547.

(11) Stier, A. V.; Wilson, N. P.; Velizhanin, K. A.; Kono, J.; Xu, X.; Crooker, S. A. Magnetooptics of Exciton Rydberg States in a Monolayer Semiconductor. *Phys. Rev. Lett.* **2018**, *120*, 057405.

(12) Birey, H. Thickness dependence of the dielectric constant and resistance of Al2O3 films. *Journal of Applied Physics* **1977**, *48*, 5209–5212.

(13) Peimyoo, N.; Barnes, M. D.; Mehew, J. D.; Sanctis, A. D.; Amit, I.; Escolar, J.; Anastasiou, K.; Rooney, A. P.; Haigh, S. J.; Russo, S.; Craciun, M. F.; Withers, F. Laser-writable high-k dielectric for van der Waals nanoelectronics. *Science Advances* **2019**, *5*, eaau0906.

(14) Van der Donck, M.; Peeters, F. M. Interlayer excitons in transition metal dichalcogenide heterostructures. *Phys. Rev. B* **2018**, *98*, 115104.

(15) Rasmussen, F. A.; Thygesen, K. S. Computational 2D materials database: electronic structure of transition-metal dichalcogenides and oxides. *The Journal of Physical Chemistry C* **2015**, *119*, 13169–13183.

(16) Pedersen, T. G.; Latini, S.; Thygesen, K. S.; Mera, H.; Nikolić, B. K. Exciton ionization in multilayer transition-metal dichalcogenides. *New Journal of Physics* **2016**, *18*, 073043.

(17) Kumar, A.; Ahluwalia, P. Tunable dielectric response of transition metals dichalcogenides MX2 (M= Mo, W; X= S, Se, Te): Effect of quantum confinement. *Physica B: Condensed Matter* **2012**, *407*, 4627–4634.

(18) Zhang, X.-X.; Cao, T.; Lu, Z.; Lin, Y.-C.; Zhang, F.; Wang, Y.; Li, Z.; Hone, J. C.;

Robinson, J. A.; Smirnov, D.; others Magnetic brightening and control of dark excitons in monolayer WSe 2. *Nature nanotechnology* **2017**, *12*, 883–888.

(19) Molas, M. R.; Faugeras, C.; Slobodeniuk, A. O.; Nogajewski, K.; Bartos, M.; Basko, D.; Potemski, M. Brightening of dark excitons in monolayers of semiconducting transition metal dichalcogenides. *2D Materials* **2017**, *4*, 021003.

(20) Zinkiewicz, M.; Grzeszczyk, M.; Kipczak, Ł.; Kazimierczuk, T.; Watanabe, K.; Taniguchi, T.; Kossacki, P.; Babiński, A.; Molas, M. R. The effect of dielectric environment on the brightening of neutral and charged dark excitons in WSe2 monolayer. *Applied Physics Letters* **2022**, *120*, 163101.

(21) Molas, M. R.; Slobodeniuk, A. O.; Kazimierczuk, T.; Nogajewski, K.; Bartos, M.; Kapuściński, P.; Oreszczuk, K.; Watanabe, K.; Taniguchi, T.; Faugeras, C.; Kossacki, P.; Basko, D. M.; Potemski, M. Probing and Manipulating Valley Coherence of Dark Excitons in Monolayer WSe_2. *Phys. Rev. Lett.* **2019**, *123*, 096803.

(22) Chen, S.-Y.; Goldstein, T.; Taniguchi, T.; Watanabe, K.; Yan, J. Coulomb-bound four- and five-particle intervalley states in an atomically-thin semiconductor. *Nature Communications* **2018**, *9*, 3717.

(23) Wang, G.; Robert, C.; Glazov, M. M.; Cadiz, F.; Courtade, E.; Amand, T.; Lagarde, D.; Taniguchi, T.; Watanabe, K.; Urbaszek, B.; Marie, X. In-Plane Propagation of Light in Transition Metal Dichalcogenide Monolayers: Optical Selection Rules. *Phys. Rev. Lett.* **2017**, *119*, 047401.

(24) Yang, M.; Ren, L.; Robert, C.; Van Tuan, D.; Lombez, L.; Urbaszek, B.; Marie, X.; Dery, H. Relaxation and darkening of excitonic complexes in electrostatically doped monolayer WSe_2: Roles of exciton-electron and trion-electron interactions. *Phys. Rev. B* **2022**, *105*, 085302.

(25) Robert, C.; Amand, T.; Cadiz, F.; Lagarde, D.; Courtade, E.; Manca, M.; Taniguchi, T.; Watanabe, K.; Urbaszek, B.; Marie, X. Fine structure and lifetime of dark excitons in transition metal dichalcogenide monolayers. *Phys. Rev. B* **2017**, *96*, 155423.

(26) Liu, E.; van Baren, J.; Liang, C.-T.; Taniguchi, T.; Watanabe, K.; Gabor, N. M.; Chang, Y.-C.; Lui, C. H. Multipath Optical Recombination of Intervalley Dark Excitons and Trions in Monolayer WSe_2. *Phys. Rev. Lett.* **2020**, *124*, 196802.

(27) Liu, E.; van Baren, J.; Lu, Z.; Altaiary, M. M.; Taniguchi, T.; Watanabe, K.; Smirnov, D.; Lui, C. H. Gate Tunable Dark Trions in Monolayer WSe_2. *Phys. Rev. Lett.* **2019**, *123*, 027401.

(28) Liu, E.; van Baren, J.; Taniguchi, T.; Watanabe, K.; Chang, Y.-C.; Lui, C. H. Valley-selective chiral phonon replicas of dark excitons and trions in monolayer WSe_2. *Phys. Rev. Res.* **2019**, *1*, 032007.

(29) Jadczak, J.; Glazov, M.; Kutrowska-Girzycka, J.; Schindler, J. J.; Debus, J.; Ho, C.-H.; Watanabe, K.; Taniguchi, T.; Bayer, M.; Bryja, L. Upconversion of Light into Bright Intravalley Excitons via Dark Intervalley Excitons in hBN-Encapsulated WSe2 Monolayers. *ACS Nano* **2021**, *15*, 19165–19174, PMID: 34735768.

(30) Zhou, Y.; Scuri, G.; Wild, D. S.; High, A. A.; Dibos, A.; Jauregui, L. A.; Shu, C.; De Greve, K.; Pistunova, K.; Joe, A. Y.; others Probing dark excitons in atomically thin semiconductors via near-field coupling to surface plasmon polaritons. *Nature nanotechnology* **2017**, *12*, 856–860.

(31) He, M.; Rivera, P.; Van Tuan, D.; Wilson, N. P.; Yang, M.; Taniguchi, T.; Watanabe, K.; Yan, J.; Mandrus, D. G.; Yu, H.; Dery, H.; Yao, W.; Xu, X. Valley phonons and exciton complexes in a monolayer semiconductor. *Nature Communications* **2020**, *11*, 618.

(32) Ye, Z.; Waldecker, L.; Ma, E. Y.; Rhodes, D.; Antony, A.; Kim, B.; Zhang, X.-X.; Deng, M.; Jiang, Y.; Lu, Z.; Smirnov, D.; Watanabe, K.; Taniguchi, T.; Hone, J.;

Heinz, T. F. Efficient generation of neutral and charged biexcitons in encapsulated WSe2 monolayers. *Nature Communications* **2018**, *9*, 3718.

(33) Barbone, M.; Montblanch, A. R.-P.; Kara, D. M.; Palacios-Berraquero, C.; Cadore, A. R.; De Fazio, D.; Pingault, B.; Mostaani, E.; Li, H.; Chen, B.; Watanabe, K.; Taniguchi, T.; Tongay, S.; Wang, G.; Ferrari, A. C.; Atatüre, M. Charge-tuneable biexciton complexes in monolayer WSe2. *Nature Communications* **2018**, *9*, 3721.

(34) Kapuściński, P.; Delhomme, A.; Vaclavkova, D.; Slobodeniuk, A. O.; Grzeszczyk, M.; Bartos, M.; Watanabe, K.; Taniguchi, T.; Faugeras, C.; Potemski, M. Rydberg series of dark excitons and the conduction band spin-orbit splitting in monolayer WSe2. *Communications Physics* **2021**, *4*, 186.

(35) Borghardt, S.; Tu, J.-S.; Taniguchi, T.; Watanabe, K.; Kardynał, B. E. Interplay of excitonic complexes in p-doped WSe_2 monolayers. *Phys. Rev. B* **2020**, *101*, 161402.

(36) Li, Z.; Wang, T.; Lu, Z.; Khatoniar, M.; Lian, Z.; Meng, Y.; Blei, M.; Taniguchi, T.; Watanabe, K.; McGill, S. A.; Tongay, S.; Menon, V. M.; Smirnov, D.; Shi, S.-F. Direct Observation of Gate-Tunable Dark Trions in Monolayer WSe2. *Nano Letters* **2019**, *19*, 6886–6893, PMID: 31487988.

(37) Li, Z.; Wang, T.; Lu, Z.; Jin, C.; Chen, Y.; Meng, Y.; Lian, Z.; Taniguchi, T.; Watanabe, K.; Zhang, S.; Smirnov, D.; Shi, S.-F. Revealing the biexciton and trion-exciton complexes in BN encapsulated WSe2. *Nature Communications* **2018**, *9*, 3719.

(38) Olsen, T.; Latini, S.; Rasmussen, F.; Thygesen, K. S. Simple Screened Hydrogen Model of Excitons in Two-Dimensional Materials. *Phys. Rev. Lett.* **2016**, *116*, 056401.

(39) Perdew, J. P.; Burke, K.; Ernzerhof, M. Generalized Gradient Approximation Made Simple. *Phys. Rev. Lett.* **1996**, *77*, 3865–3868.
